# A DFT Perspective on Cd^2+^ Removal by CaCO_3_/FAU Zeolite Frameworks: Bridging Theory and Experiment

**DOI:** 10.3390/jox16040133

**Published:** 2026-07-22

**Authors:** Ramon S. da Silva, Mendelssolm K. Pietre, Rodrigo G. Amorim

**Affiliations:** 1 Departamento de Física, Instituto de Ciências Exatas—ICE, Universidade Federal de Juiz de Fora, Campus Universitário, Juiz de Fora 36036-900, MG, Brazil; 2 Departamento de Química, Instituto de Ciências Exatas—ICEx, Universidade Federal Fluminense, Volta Redonda 27213-415, RJ, Brazil; 3 Departamento de Física, Instituto de Ciências Exatas—ICEx, Universidade Federal Fluminense, Volta Redonda 27213-415, RJ, Brazil; rgamorim@id.uff.br

**Keywords:** faujasite, DFT calculation, ion exchange process

## Abstract

The efficient removal of toxic heavy metals from wastewater remains a critical environmental challenge. Faujasite (FAU) zeolite is highly effective for this purpose due to its high surface area, microporous structure, and exceptional ion exchange capacity. This study employs density functional theory (DFT) calculations to investigate the thermodynamics and geometry of ${\mathrm{Cd}}^{2+}$ capture by two distinct processes: (i) ion exchange and (ii) ${\mathrm{CaCO}}_{3}$/FAU adsorption. We systematically identify preferential binding sites and calculate binding energies for ${\mathrm{Cd}}^{2+}$ within the FAU framework. As a key result, the current theoretical modeling confirms previous experimental observations that dispersed ${\mathrm{CaCO}}_{3}$ units on the surface of faujasite enhance ${\mathrm{Cd}}^{2+}$ adsorption compared to aggregated units. The computed binding energies for the dispersed configuration range from −54 kcal/mol to −60 kcal/mol, depending on the number of ${\mathrm{CaCO}}_{3}$ units. These findings provide a fundamental theoretical perspective and represent a first step toward understanding ${\mathrm{Cd}}^{2+}$ removal by modified zeolites.

## 1. Introduction

The increasing contamination of water sources by heavy metals such as lead (${\mathrm{Pb}}^{2+}$), cadmium (${\mathrm{Cd}}^{2+}$), copper (${\mathrm{Cu}}^{2+}$), and zinc (${\mathrm{Zn}}^{2+}$) poses a significant threat to environmental and public health due to their toxicity, persistence, and bioaccumulation [1,2,3]. Traditional methods for heavy metal removal, including chemical precipitation, membrane filtration, and ion exchange, often suffer from limitations such as high operational costs, sludge generation, and low selectivity.

In recent years, zeolites have emerged as promising adsorbent materials for the efficient removal of heavy metals from aqueous solutions [4,5,6,7]. Zeolites are crystalline aluminosilicates with a well-defined porous structure [8,9], high cation exchange capacity [10], and strong affinity for various metal ions [8]. Their abundance, low cost, especially in the case of natural zeolites, and ease of regeneration make them highly attractive for large-scale water treatment applications [11].

In particular, faujasite stands out due to its high surface area (>500 $m^{2}$/g) [12], high Al content (low Si/Al molar ratio) [13], and pore size and cavities accessible by the main toxic metal ions [14]. Furthermore, its synthesis is conducted without the presence of organic molecules, making the process eco-friendly, in line with the principles of green chemistry [15].

From a theoretical perspective, several models describing the structural parameters of zeolites have been proposed in the literature [8,16,17,18,19,20]. For faujasite, their structure consists of a tetrahedral (T-O, T = Si, Al) framework forming large supercages accessible through 12-membered ring windows. The isomorphous substitution of ${\mathrm{Si}}^{4+}$ by ${\mathrm{Al}}^{3+}$ introduces a negative charge on the framework, which is typically balanced by extra-framework cations such as ${\mathrm{Na}}^{+}$. The location and coordination of these cations are crucial for the material’s properties.

A primary application of these models has been to study the adsorption of pollutant gases, including carbon oxides (CO and ${\mathrm{CO}}_{2}$), nitrogen monoxide (NO), and nitrogen dioxide (${\mathrm{NO}}_{2}$). For instance, Daouli et al. [16] employed periodic density functional theory (DFT) calculations to investigate the interactions between ${\mathrm{NO}}_{x}$ contaminants and faujasites doped with a series of divalent cations (${\mathrm{Be}}^{2+}$, ${\mathrm{Mg}}^{2+}$, ${\mathrm{Ca}}^{2+}$, ${\mathrm{Sr}}^{2+}$, ${\mathrm{Ba}}^{2+}$, ${\mathrm{Fe}}^{2+}$, ${\mathrm{Cu}}^{2+}$, ${\mathrm{Zn}}^{2+}$, ${\mathrm{Pd}}^{2+}$, and ${\mathrm{Pt}}^{2+}$). Using a Si/Al ratio of 2.4, they concluded that ${\mathrm{Pd}}^{2+}$ and ${\mathrm{Pt}}^{2+}$ cations significantly enhance ${\mathrm{NO}}_{x}$ adsorption within the faujasite framework. In general, the Si/Al ratio provides insight into the charge density of the zeolite framework; a lower ratio indicates more aluminum atoms, more negative charges to balance and, consequently, a higher capacity for cation exchange. Similarly, Hessou and coworkers [18] employed a comparable strategy to examine the role of monovalent cations (${\mathrm{Li}}^{+}$, ${\mathrm{Na}}^{+}$, $K^{+}$, ${\mathrm{Rb}}^{+}$, ${\mathrm{Cs}}^{+}$, ${\mathrm{Ag}}^{+}$, and ${\mathrm{Cu}}^{+}$) on the adsorption of ${\mathrm{NO}}_{x}$ and ${\mathrm{CO}}_{x}$ gases.

Karamanis et al. [8] investigated a novel adsorption model for harmful gases and water by exploring different Si/Al ratios of 1.4, 2.43, 23, and 47. Karamanis et al. [8] demonstrated that lower Si/Al ratios (e.g., X zeolite, Si/Al = 1.4) lead to stronger adsorption energies for NO, ${\mathrm{NO}}_{2}$, and H_2_O due to higher cation density and cross-adsorption between sites II and III′. However, X zeolites are significantly more hydrophilic, making them less selective for NOx over H_2_O, especially for alkaline cations. On the other hand, Chiu et al. [17] also employed periodic DFT calculations to determine accurate adsorption energies of water, alkanes, and alcohols in silicalite and HZSM-5 zeolites. A non-periodic quantum chemical approach, combining the semiempirical AM1 method with the hybrid density functional B3LYP/6-31G method, was employed to investigate the acid sites in Zeolite MCM-22 [21].

Moving beyond standard DFT approaches, Goncalves et al. [22] performed single-point calculations with high-level wavefunction methods—namely, Möller–Plesset perturbation theory (MP2) and non-canonical coupled cluster theory—in combination with a larger basis set from the Ahlrichs group [23] to study zeolite catalysis. In the context of hydrogen storage, researchers such as Deeg et al. [24] and Kang et al. [25] have explored the capacity of zeolites for this application. The catalytic cycloaddition of ethylene oxide and carbon dioxide to form ethylene carbonate on M(II)-faujasite zeolites (M = Ni, Cu, Zn) was investigated using the Minnesota M06-L meta-GGA functional [26].

The process of ion exchange in zeolites is, however, a complex phenomenon governed by a delicate interplay of thermodynamic and structural factors [19,27,28]. It is not a simple substitution but a sophisticated competition influenced by the charge and hydration energy of the incoming cation [29], the structure and charge density of the zeolite framework, and the dynamics of cation dehydration and diffusion [30]. Consequently, numerous studies have been dedicated to developing a more complete understanding of this process. For instance, Sellaoui et al. [19] studied the adsorption mechanism of faujasite-type zeolite Y for heavy metals such as ${\mathrm{Ag}}^{+}$, ${\mathrm{Cu}}^{2+}$, and ${\mathrm{Co}}^{2+}$ using periodic DFT calculations. Their results demonstrated a preference for ${\mathrm{Ag}}^{+}$ over the other metals studied.

Monte Carlo simulations were conducted by Jeffroy and coworkers [31] with a Si/Al ratio equal 2.69 to gain a deeper understanding of the ion exchange behavior of sodium in NaY faujasite with alkali metals: lithium, potassium, rubidium, and cesium.

**Figure 1:**
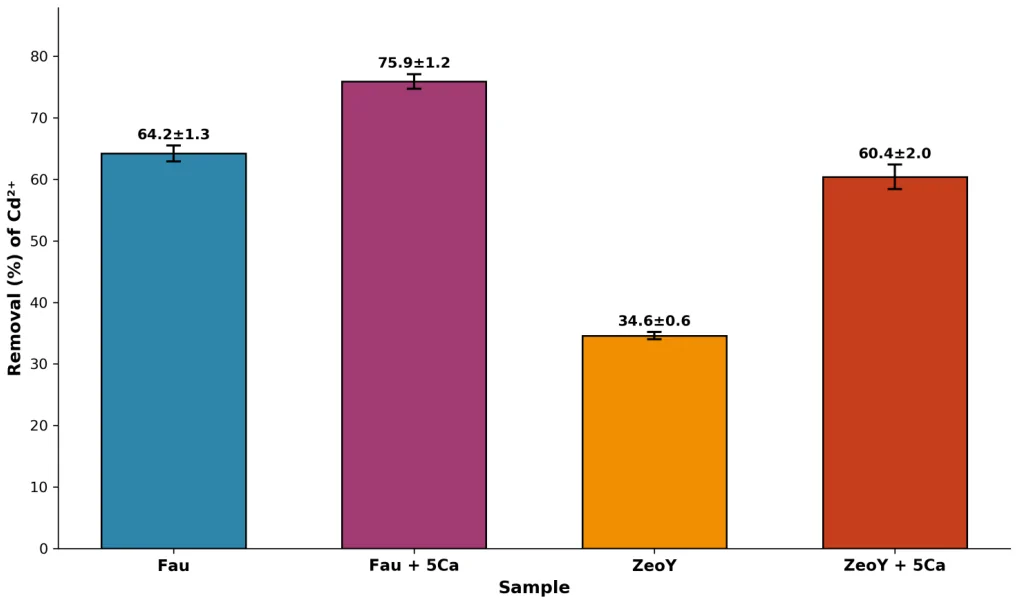
Experimental results regarding the removal percentages of Cadmium ions utilizing four distinct adsorbent materials.

Figure 1 shows the efficiency of ${\mathrm{Cd}}^{2+}$ removal from aqueous solution using synthetic FAU-type zeolites investigated experimentally by Profeta et al. [32], who proposed two different mechanisms. In the first one, the authors found that modifying the zeolite by dispersing calcium carbonate (${\mathrm{CaCO}}_{3}$) particles on its surface improved its cadmium removal performance. Their work also indicated that ion exchange was the primary mechanism for ${\mathrm{Cd}}^{2+}$ capture, which provides a foundation for designing more efficient zeolite-based adsorbents. In addition, Profeta and coworkers observed a possible ion exchange occurring between “free” ${\mathrm{Na}}^{+}$ ions on the surface and ${\mathrm{Cd}}^{2+}$ ions.

This work aims to use a theoretical model for an FAU/${\mathrm{CaCO}}_{3}$ composite, investigated through density functional theory, to elucidate the main factors governing the ion exchange process. The focus is on understanding how dispersed calcium carbonate particles on the zeolite surface contribute to improved performance in cadmium ion removal.

## 2. Materials and Methods

### 2.1. Computational Method

All quantum chemistry calculations were performed using the ORCA suite of programs [33,34]. To investigate the ion exchange process, we perform density functional theory (DFT) calculations employing the hybrid B3LYP functional [35,36] in combination with def2-TZVPP basis set [23], generally contracted to [8s6p4d3f1g/_C,O_;-9s7p6d5f2g/_Si,Al_;11s8p7d5f/_Ca_;8s7p6d5f3g2h/_Cd_ 4s2p1d/_H_], and the corresponding auxiliary def2-TZVP/C basis set. Self-consistent field (SCF) interactions converged to $10^{-9}$ $E_{h}$ in the total energy using the *VeryTightSCF* criterion. The Grimme’s D3 dispersion correction [37] (with Becke–Johnson damping [38]) was also included on the optimized molecular geometries to accurately model the dispersion component of non-covalent interactions.

### 2.2. Surface Model

**Figure 2:**
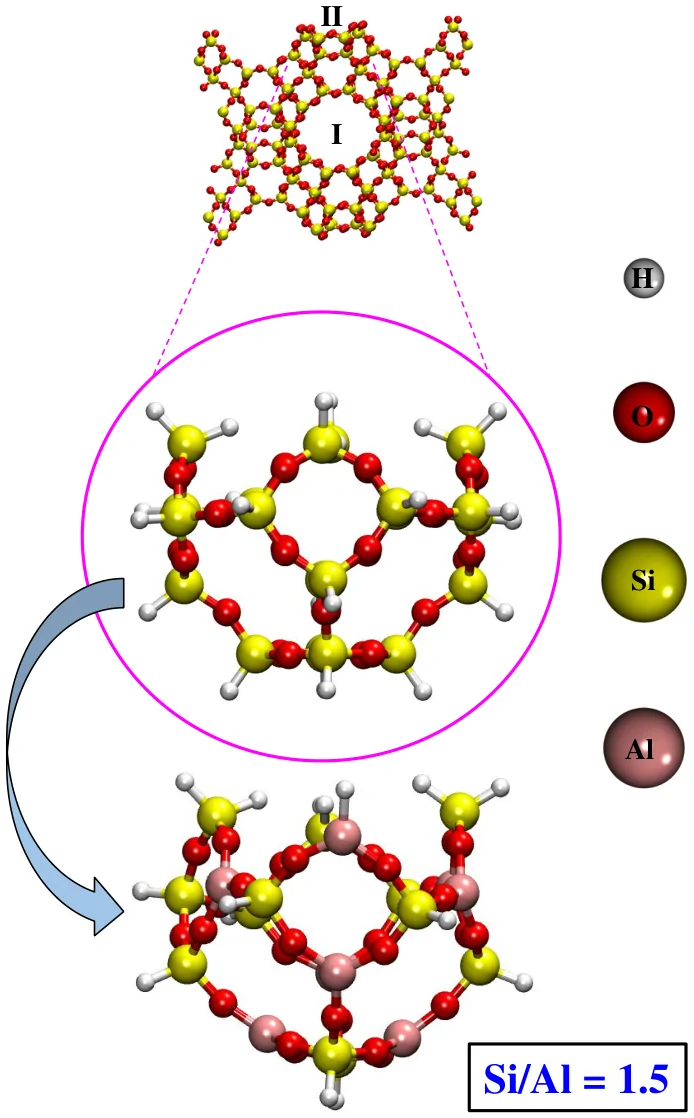
Structural model of the faujasite zeolite fragment employed in this work, showing the optimized cage structure at site II. The final model corresponds to a Si/Al ratio of 1.5 in line with experimental observations [32].

In Figure 2, the purely siliceous faujasite framework is depicted, consisting of a total of 576 atoms, distributed as 192 silicon and 384 oxygen atoms. For the present study, a cluster model of the faujasite zeolite was constructed to reproduce a cage (site II) close to the supercage structure (site I), as highlighted in the same figure. The resulting cluster, with stoichiometry ${\mathrm{Al}}_{8}$${\mathrm{Si}}_{12}$$O_{24}$$H_{10}$ and comprising 64 atoms, was employed to reduce the computational cost associated with DFT calculations. Hydrogen atoms were introduced to saturate the dangling bonds at the cluster boundaries (no Brønsted acid sites). A Si/Al ratio of ∼1.5 was chosen to enable a direct comparison with our recent experimental investigation by Profeta et al. [32]. The resulting structure is presented in Figure 2, along with the corresponding atom labeling.

The modeled structure primarily consists of four 12-membered rings, each connected to four 8-membered rings. In accordance with the Loewenstein rule [39], two aluminum atoms are prevented from sharing an oxygen bridge (Al-O-Al) at neighboring tetrahedral sites, since this configuration is energetically unfavorable. In essence, Al-O-Al linkages are forbidden, as they would require a common oxygen atom to be shared between two adjacent ${\mathrm{AlO}}_{4}$ tetrahedra. The fundamental building block of the faujasite structure is the ${\mathrm{TO}}_{4}$ tetrahedron. A statistical analysis of the bond lengths and bond angles in the provided model was conducted. The results are summarized in Table 1.

The accurate determination of tetrahedral bond lengths in zeolites and other aluminosilicates is fundamental for understanding their crystal chemistry and ion exchange properties. An extensive compilation of experimental studies by Smith and Bailey [40] established the quantitative relationships between Si-O and Al-O distances and their dependence on structural factors. According to them, the primary difference between Si-O and Al-O distances arises from the disparity in ionic radii: Al^3+^ in tetrahedral coordination (0.39 Å) is considerably larger than Si^4+^ (0.26 Å) [41]. Consequently, Al-O bonds are systematically longer than Si-O bonds. Smith and Bailey demonstrated that the mean tetrahedral distance 〈T-O〉 increases linearly with aluminum content for a given structural type, but the absolute values depend critically on the degree of tetrahedral polymerization.

For framework structures (tectosilicates, O/T = 2), where all oxygens are shared between tetrahedra, the mean Si-O distance is approximately 1.61 Å. Extrapolation to pure Al yields a mean Al-O distance of 1.75 Å. These values are confirmed by ordered structures such as anorthite (${\mathrm{CaAl}}_{2}$${\mathrm{Si}}_{2}$$O_{8}$), where individual Al-O tetrahedra average 1.749 Å. For sheet structures (phyllosilicates, O/T ≈ 2.5), the corresponding values are slightly larger: Si-O ≈ 1.62 Å and Al-O ≈ 1.77 Å. For isolated tetrahedra (nesosilicates, O/T = 4), the distances increase further to Si-O ≈ 1.63 Å and Al-O ≈ 1.80 Å [40].

The present Si-O bond distances range from 1.66 to 1.68 Å, in good agreement with the reference value of 1.638 Å reported by Song et al. [42]. Uzunova and Nikolov [43], who investigated the geometries of neutral and ionic zeolite clusters using DFT, reported Si-O bond lengths of approximately 1.64–1.65 Å. Periodic DFT calculations by Astala and coworkers [44] yielded a slightly shorter average Si-O bond distance of 1.60 Å. In the present work, the average Al-O bond distance is 1.712 Å, which is marginally shorter than the values of 1.77–1.79 Å reported by Uzunova and Nikolov. The O-Si-O bond angles range from 108° to 109°, consistent with the findings of Uzunova and Nikolov. Similar trends were reported by Uzunova and Mikosch [45] and Astala et al. [44]. Our calculated average O-Al-O and Al-O-Si bond angles are 119.6° and 167.5°, respectively.

Comparison with the experimental compilation of Smith and Bailey [40] shows that our calculated Si-O distance is slightly higher than the value reported for framework silicates, while our Al-O distance is slightly lower than the experimental reference (1.75 Å). This minor deviation is consistent with the known tendency of the B3LYP functional and cluster models to slightly overestimate Si-O and underestimate Al-O bond lengths in ionic systems.

**Figure 3:**
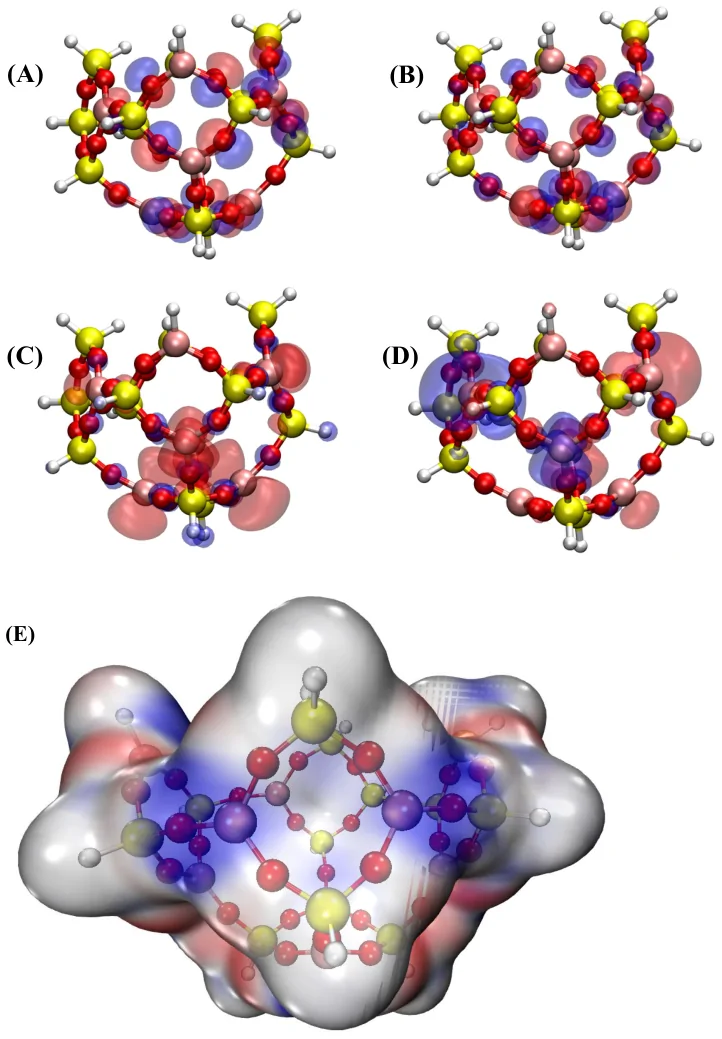
Molecular orbital visualization: (**A**) HOMO-1, (**B**) HOMO, (**C**) LUMO, (**D**) LUMO+1, and (**E**) Molecular Electrostatic Potentials (MEP) with atoms colored by element: Silicon (yellow), Oxygen (red), Aluminum (pink), and Hydrogen (white). Positive phase isosurfaces are shown in blue, negative phase in red. The isosurface value was set to 0.02 atomic units for optimal visualization of nodal patterns.

Figure 3 presents the equilibrium geometry molecular orbitals and molecular electrostatic potentials (MEP) of the system under study. The orbital surfaces show that the highest occupied molecular orbital (HOMO, Figure 3B) is predominantly localized over electron-rich regions, particularly the oxygen atoms within the π-system. The computed HOMO and lowest unoccupied molecular orbital (LUMO) energies are −7.99 eV and −1.55 eV, respectively, yielding a HOMO–LUMO gap (ΔE) of 6.44 eV. This value lies within the 5.46–9.14 eV range predicted by Uzunova and Mikosch [45] for FAU zeolites and is also consistent with the 5.5–8.2 eV interval reported by Uzunova and Nikolov [43]. However, it deviates notably from the ∼2.7 eV gap reported by Jardillier et al. [46] for CuY zeolite.

Since computed data for the pristine analogue of the present system are not readily available in the literature, the obtained gap was benchmarked against selected literature values for related zeolite clusters. The results are presented in Table 2. The computed gap of 6.44 eV (B3LYP-D3/def2-TZVPP) falls within the 6.24–6.95 eV range reported for Be-, Mg-, and Ca-substituted ERI clusters [47], with the closest match to the Mg-ERI value (6.52 eV). The minor discrepancies are attributable to differences in the exchange–correlation functional and basis set. Notably, this gap is substantially narrower than the ∼9.1 eV gaps typical of purely siliceous models [45], yet remains considerably wider than the 1.36–2.62 eV range characteristic of transition-metal-doped zeolites, where partially filled *d* orbitals introduce pronounced mid-gap states [48]. Collectively, these comparisons situate the present system in an intermediate electronic regime, closer to alkaline-earth-substituted aluminosilicates than to metal-functionalized or fully insulating frameworks.

Finally, the MEP map in Figure 3E shows high electron density (negative electrostatic potential) concentrated around the ring oxygen atoms (red regions), while the most positive areas correspond to the hydrogen atoms (blue zones). This charge distribution is fully consistent with the electropositive nature of hydrogen.

## 3. Results and Discussion

### 3.1. Relative Stability of ${\mathrm{Na}}^{+}$ Adsorption Sites

**Figure 4:**
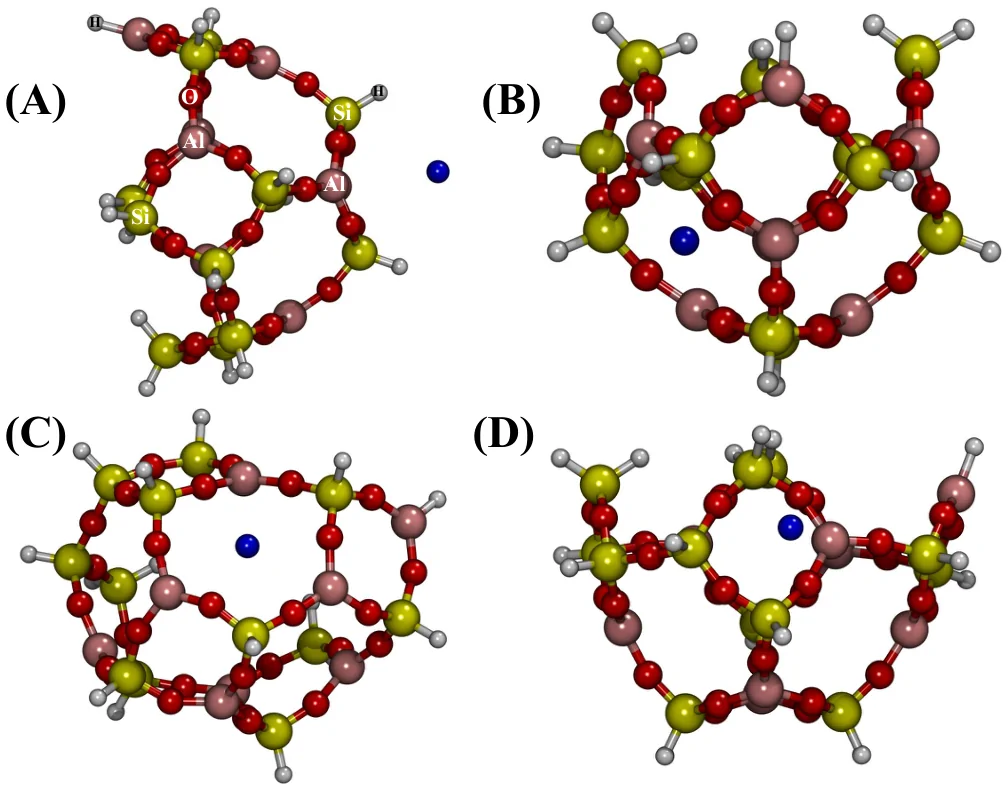
Optimized structures of a ${\mathrm{Na}}^{+}$ cation (blue) adsorbed on the faujasite surface at four distinct sites: (**A**) **Site 1**, located at an 8-membered ring containing two Al atoms (highlighted in pink); (**B**) **Site 2**, located at a 12-membered ring containing two Al atoms; (**C**) **Site 3**, located at a 12-membered ring containing three Al atoms; and (**D**) **Site 4**, located near an 8-membered ring containing a single Al atom. The aluminum atoms are shown in pink for clarity. See also Supplementary Information.

To identify the most stable adsorption site for ${\mathrm{Na}}^{+}$ in the FAU zeolite, we compared the total energies of four distinct atomic configurations, as depicted in Figure 4. The corresponding energy differences (ΔE) and binding energies (BE) are summarized in Table 3, with ΔE referenced to the lowest-energy structure. **Site 1** is located at an 8-membered ring with two Al atoms, **Site 2** at a 12-membered ring with two Al atoms, and **Site 3** at a 12-membered ring with three Al atoms. **Site 4** is positioned near an 8-membered ring with a single Al atom, as shown in Figure 4. See also Figure S1 of the Supplementary Information (SI).

We calculated the binding energies (BE) using the following equation:

(1) $$\mathrm{BE}=E\left(\text{Na–FAU}\;\mathrm{complex}\right)-\left[E(\mathrm{FAU})+E(\mathrm{Na})\right]$$

where E (FAU) represents the energy of the clean faujasite, E(Na) is the energy of the isolated alkali metal, and E(Na–FAU complex) denotes the energy of FAU with adsorbed atom.

The results demonstrate a clear hierarchy of stability among the sites. The most stable configuration corresponds to **Site 2** (ΔE = 0.0 kcal/mol; BE = −21.89 kcal/mol), followed very closely by **Site 3**, with a small energy difference of only 0.57 kcal/mol. This small value suggests that both sites may be populated under experimental conditions. In contrast, **Sites 1 and 4** exhibit substantially larger energy differences (13.50 and 18.58 kcal/mol, respectively), indicating they are significantly less favorable configurations. The negative binding energy values for all sites confirm that the interaction between the ${\mathrm{Na}}^{+}$ cation and the FAU zeolite framework is energetically favorable.

**Figure 5:**
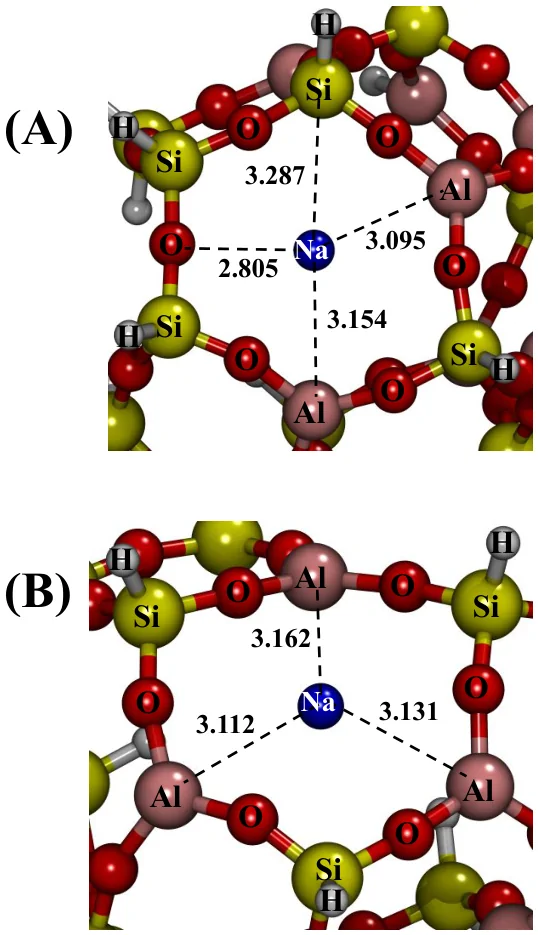
Optimized structures of a ${\mathrm{Na}}^{+}$ cation (blue) adsorbed on the faujasite surface at two distinct sites: (**A**) a 12-membered ring containing two Al atoms, and (**B**) a 12-membered ring containing three Al atoms.

Figure 5 presents the structural details of the two most stable adsorption sites: **Site 2** and **Site 3**. This observed strong correlation is attributed to fact that each aluminum atom in the FAU framework introduces a localized negative charge, which is compensated by a cation via a strong, predominantly electrostatic interaction. The superior stability of these sites confirms that a higher density of anionic centers enhances ${\mathrm{Na}}^{+}$ binding. We identify average r(Al–Na) bond distances of approximately 3.124 Å and 3.135 Å for **Site 2** and **Site 3**, respectively. In **Site 2**, the r(Na–O) bond lengths within the twelve-membered ring range from 2.567 Å to 3.204 Å. DFT/LDA studies conducted by Vayssilov et al. [52], focusing on the twelve-membered ring, reported Na–O distances in the range of 2.18–2.47 Å. In a complementary approach, Torres et al. [53] investigated smaller cluster models using different basis sets consisting of four-, five-, and six-membered rings, saturating the silicon and aluminum dangling bonds with hydrogen atoms [(Si_*n*−1_AlO_*n*_H_2*n*_)^−^, with *n* = 4, 5, 6]. Their calculations yielded r(Na–O) distances of 2.642–2.303 Å for the four-membered ring, 2.282–2.804 Å for the five-membered ring, and 2.262–3.173 Å for the six-membered ring, in line with our findings.

A direct comparison between **Site 1** and **Site 2**, which both possess two aluminum atoms, emphasizes the significant influence of local geometry. The drastic energy difference of 13.50 kcal/mol strongly suggests that the 12-membered ring (**Site 2**) provides a far more favorable coordination environment for the ${\mathrm{Na}}^{+}$ cation than the 8-membered ring (**Site 1**, Figure 4A).

The very low binding energy of **Site 4**, which is associated with only a single aluminum atom, underscores a fundamental requirement for strong ${\mathrm{Na}}^{+}$ adsorption. We can conclude that a solitary anionic site generates an insufficient electrostatic potential to stabilize the cation effectively. The resulting weak binding energy of −3.30 kcal/mol confirms that such sites are not significant for cation retention under typical conditions.

These findings are consistent with previous computational studies on related zeolitic systems. For instance, Vorontsov et al. [54] demonstrated that in mordenite, the specific location of Al atoms at distinct tetrahedral sites (T1–T4) significantly influences both the stability of active sites and the strength of adsorbate interactions. Based on the energetic comparison of the adsorption sites, the atomic structure shown in **Site 2** [Figure 4B] was selected as the reference for subsequent ion exchange studies, as it provides a representative environment for the reaction.

Compared to other ${\mathrm{Na}}^{+}$ adsorbents, the Linde Type A (LTA) zeolite, a promising material for radioactive decontamination processes studied by Kocevski et al. [55] via periodic DFT calculations, shows related performance. They reported ${\mathrm{Na}}^{+}$ adsorption energies on the LTA surface in water solvent, ranging from 33 to 46 kcal/mol, in qualitative agreement with the present findings. Separately, the zeolite faujasite structure examined by Chatterjee and Mizukami [56] yielded an adsorption energy of –15.68 kcal/mol, which aligns with our results. However, the current data differ from those in the earlier study by Chu and coworkers [57], who reported adsorption energies of approximately 120 kcal/mol.

While Karamanis et al. [8] reported Na^+^ binding energies on Y zeolite of approximately −5.9 kcal/mol, our cluster model yields a value of −21.89 kcal/mol for the most stable site (Site II). This quantitative difference is attributed to the cluster approximation and the different exchange–correlation functionals employed, but the relative stability order of adsorption sites remains consistent across both studies, validating the use of our cluster model for comparative analysis.

### 3.2. Proposed Model for Ion Exchange of ${\mathrm{Na}}^{+}$ by ${\mathrm{Cd}}^{2+}$ on the FAU Surface

From a thermodynamic point of view, both endothermic and exothermic ion exchange reactions were experimentally observed by Barros et al. [58] while investigating the interactions between $K^{+}$, ${\mathrm{Ca}}^{2+}$, and ${\mathrm{Cr}}^{3+}$ ions in Zeolite NaA. According to their results, positive enthalpy values for the K–NaA exchange were found 6.44 kcal/mol and 2.27 kcal/mol at 30 and 45 °C. Elwakeel and co-workers [59] reported that the adsorption of Cu(II) and Cd(II) ions onto APNa-Y zeolite was endothermic, whereas Pb(II) recovery was exothermic. In another study, Elwakeel et al. [60] demonstrated that the adsorption of Cu(II) and Cd(II) ions from aqueous solution onto a methyl methacrylate–Na-Y zeolite (MMA–Na-Y zeolite) composite was also endothermic. Recently, the adsorption of ${\mathrm{Cd}}^{2+}$ on chitosan-loaded natural zeolite (CNZ) was demonstrated to be an endothermic reaction (ΔH = 3.17 kcal/mol) [61].

**Figure 6:**
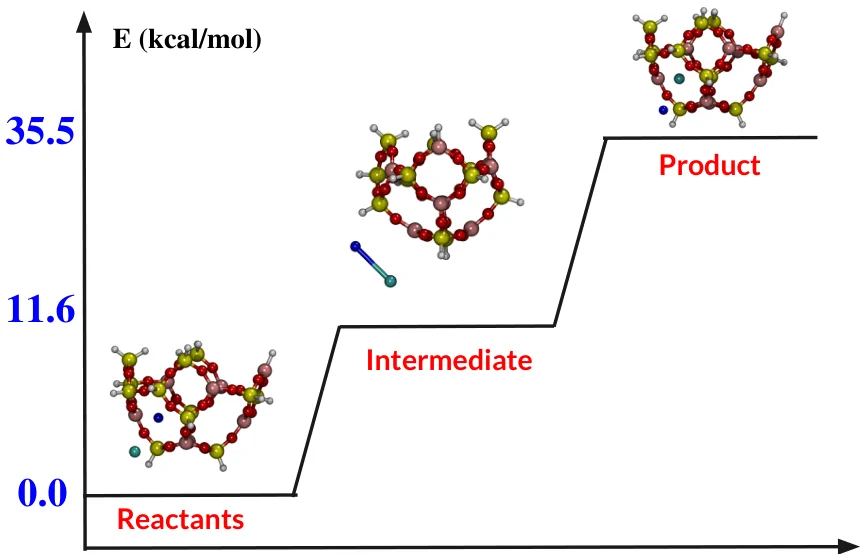
Proposed reaction pathway for ion exchange between Cd (light blue) and Na (dark blue) atoms, proceeding via the formation of an unstable NaCd intermediate species. Optimized structures were calculated at the B3LYP/def2-TZVPP level of theory.

**Figure 7:**
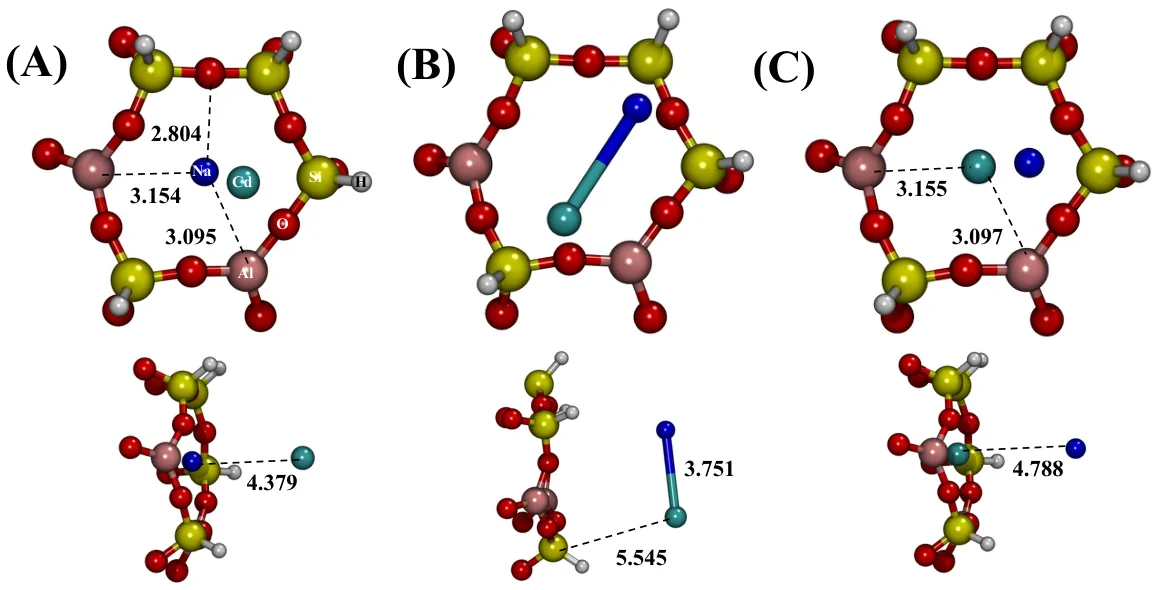
Detailed view of the ion exchange pathway between cadmium (light blue) and sodium (dark blue) atoms: (**A**) initial configuration with Cd and Na at a relatively large distance; (**B**) formation of the possible unstable NaCd intermediate species; (**C**) final configuration after ion exchange. All distances are given in Å.

Following this line of reasoning, we introduced a ${\mathrm{Cd}}^{2+}$ atom into our model at **Site 2** to evaluate the ion exchange reaction between ${\mathrm{Na}}^{+}$ and ${\mathrm{Cd}}^{2+}$. The proposed reaction pathway using the B3LYP-D3BJ/def2-TZVPP level of theory is presented in Figure 6. As can be seen, the reactants are a ${\mathrm{Cd}}^{2+}$ atom and Na–Faujasite. Figure 7A reveals that the Na-Cd bond distance r = 4.379 Å is relatively large, indicating that this interaction is predominantly long-range electrostatic in nature and can be described in terms of multipole expansions. Figure 7B shows the formation of a Na-Cd species, likely corresponding to an unstable intermediate with a presumably short lifetime. Ab initio calculations performed by Angeli and Persico [62] indicated that the NaCd($X^{2}\Sigma^{+}$) state has a dissociation energy of 0.0584 eV (471 ${\mathrm{cm}}^{-1}$) at an equilibrium bond distance of 3.316 Å. This shallow potential supports our assumption regarding the instability of the Na-Cd intermediate. Furthermore, additional B3LYP-D3BJ/def2-TZVPP calculations were performed to evaluate the molecular properties of the NaCd diatom. As a result, we found an equilibrium bond distance of 3.393 Å, a vibrational frequency of 83 ${\mathrm{cm}}^{-1}$, and a dipole moment of 0.51 D. These values qualitatively agree with those reported by Angeli and Persico.

Examination of Figure 7B reveals that the r(Na-Cd) bond distance is 3.751 Å, differing by approximately 0.358 Å from the equilibrium bond distance calculated for the free NaCd species (r = 3.393 Å). Considering the short lifetime of the NaCd ground state, the diatom is expected to rapidly dissociate through the Na(ns) + Cd(ns) dissociation channel. Figure 7C shows that the r(Na-Cd) bond distance increases from 4.379 Å in Figure 7A to 4.788 Å.

### 3.3. Atomic-Level Analysis of Cadmium Removal on the ${\mathrm{CaCO}}_{3}$/FAU Surface

The structure of calcium carbonate (${\mathrm{CaCO}}_{3}$), fundamental to our model, is now presented in detail. Its corresponding optimized geometry is shown in Figure S2 in the Supplementary Information. As illustrated, the oxygen atom is bonded to carbon at a distance of 1.206 Å, forming the C=O bond. The two other oxygen atoms are bonded to carbon at slightly longer average distance (1.357 Å). Their O-C-O bond angles are approximately 124.4°, close to the ideal trigonal planar geometry of carbonate (120°). The key structural feature is the position of the calcium atom. It is bonded to oxygen atoms at a distance of ≈2.05 Å, which is a characteristic distance for a Ca-O ionic interaction. The Ca-O-C angle is 91.5°, and the associated dihedral angle places the calcium atom out of the ${\mathrm{CO}}_{3}$ plane. We compute the O-Ca-O bond angle to be 66°. These results show excellent quantitative agreement with those of Gupta and coworkers [63], who reported r(Ca-O) = 2.07 Å, r(C-O) = 1.36 Å, and r(C=O) = 1.21 Å.

The carbon atom carries a significant positive partial charge of +0.42e, a consequence of the electron-withdrawing nature of the three oxygen atoms. The three oxygen atoms are all negatively charged. The oxygen atom constituting the carbonyl (C=O) group carries a partial charge of −0.35e. The calcium atom exhibits a large positive charge of +1.20e. This value is substantially less than its formal charge of +2e, indicating a significant transfer of electron density from the coordinating oxygen and, to a lesser extent, through-space polarization from the other atoms, towards the calcium cation. The significant molecular dipole moment of 13.3 D is a direct consequence of the combined geometric and electronic structure. This value is in excellent quantitative agreement with the B3LYP/6-311++G(d,p) value of 13.8 D reported by Gupta et al. [63]. In addition, the computed HOMO–LUMO gap of 2.89 eV is consistent with the value of 2.90 eV obtained by the same authors.

The computed harmonic vibrational frequencies for ${\mathrm{CaCO}}_{3}$ are presented in Table S1 of the Supplementary Information alongside experimental references [63,64]. The calculated frequencies generally agree well with experimental data, with deviations typically below 5%. The lowest frequency mode (118 ${\mathrm{cm}}^{-1}$) corresponds to the hindered rotation of the carbonate group, which is often challenging to measure experimentally, potentially explaining its absence in the reported literature. The high-frequency mode at 1767 ${\mathrm{cm}}^{-1}$, associated with the asymmetric C-O stretching vibration, shows excellent agreement with experimental values (1794 and 1788 ${\mathrm{cm}}^{-1}$), with a discrepancy of only 1.5%.

**Figure 8:**
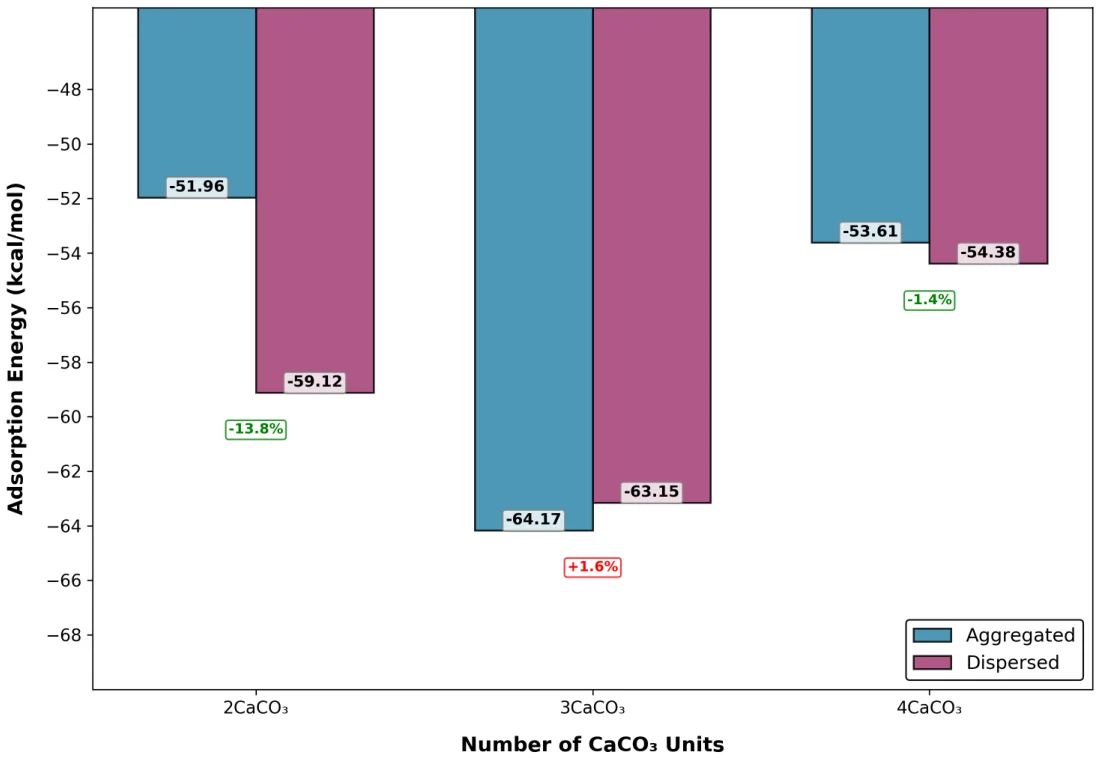
Comparison of cadmium adsorption energies for aggregated (blue) and dispersed (magenta) CaCO_3_ configurations on faujasite zeolite. More negative values indicate stronger adsorption. Dispersed configurations show enhanced adsorption energies across all CaCO_3_ loadings (2–4 units), with percentage differences between configurations indicated below each pair.

Having validated the accuracy of our ${\mathrm{CaCO}}_{3}$ model against established benchmarks, we proceeded to calculate its effect on the adsorption energy of cadmium onto FAU zeolite. The adsorption energy data presented in Figure 8 reveal significant trends in the interaction between ${\mathrm{Cd}}^{2+}$ and faujasite zeolite modified with calcium carbonate. Two distinct configurations were investigated: (i) aggregated (Figure S3) and (ii) dispersed CaCO_3_ arrangements (Figure S4). The aggregated configuration was analyzed for systems containing 1 to 4 CaCO_3_ units. In contrast, the dispersed configuration was examined only for systems with 2 to 4 CaCO_3_ units, limited due to the size of the faujasite zeolite model.

For the aggregated systems, the adsorption energy shows an overall increasing trend with the number of CaCO_3_ units, starting at −51.88 kcal/mol for the single CaCO_3_ system and reaching −75.18 kcal/mol for the system with six CaCO_3_ units. This represents a substantial 45% increase in adsorption strength across the series. The progression is not perfectly monotonic, with noticeable variations at intermediate compositions: the system with three CaCO_3_ units [Figure S3C] exhibits an adsorption energy of −64.17 kcal/mol, which decreases to −53.61 kcal/mol for the four CaCO_3_ units [Figure S3D] before increasing again. This non-linear behavior suggests possible structural rearrangements or cooperative effects that merit further investigation.

The dispersed configuration presents different adsorption characteristics. For systems containing two CaCO_3_ units [Figure S4A], the dispersed arrangement yields a more favorable adsorption energy (−59.12 kcal/mol) compared to the aggregated counterpart (−51.96 kcal/mol), representing a 13.8% enhancement. This trend continues for the three CaCO_3_ units, where the dispersed configuration shows an adsorption energy of −63.15 kcal/mol versus −64.17 kcal/mol for the aggregated form. Interestingly, at this molecular configuration, the aggregated configuration becomes slightly more favorable by approximately 1.6%. For the four CaCO_3_ units [Figure S4C], the dispersed configuration again becomes more favorable (−54.38 kcal/mol compared to −53.61 kcal/mol for aggregated), though the difference is relatively small at 1.4%.

Notable changes in charge distribution are observed for the two ${\mathrm{CaCO}}_{3}$ configuration upon interaction with the dispersed faujasite framework. Specifically, the calcium atom undergoes a reduction in positive charge from +1.20e in the isolated species to +1.07e in the complex, consistent with net electron donation. On the other hand, the carbon atoms in both adsorbed species become more positively charged (∼+0.45e to +0.48e) as compared to the isolated molecule (+0.42e).

The inclusion of a third calcium carbonate within the faujasite framework reinforces and extends the charge redistribution trends observed with two units, while introducing new extremes. The electron deficiency on carbon becomes more pronounced. Charges now range from +0.49e to +0.65e, substantially higher than in the isolated molecule. Oxygen charges exhibit an even wider distribution (−0.66e to −0.39e). In contrast, the charge on the Cd atom remains small and stable, close to 0.11e.

These results suggest that both the quantity and spatial arrangement of CaCO_3_ modifiers significantly influence ${\mathrm{Cd}}^{2+}$ adsorption on faujasite. The general enhancement of adsorption strength with increasing CaCO_3_ content implies potential synergistic effects between the zeolite framework and carbonate groups. The comparison between aggregated and dispersed configurations reveals that spatial distribution can modulate adsorption energetics by 1–14% depending on molecular configuration, suggesting that controlled synthesis of CaCO_3_ distribution could optimize adsorption performance for specific applications.

Compared with other ${\mathrm{Cd}}^{+2}$ adsorbents, the FAU/${\mathrm{CaCO}}_{3}$ configuration demonstrated superior cadmium removal performance relative to work of Aloui et al. [65], who reported an adsorption energy of −6.81 kcal/mol for ${\mathrm{Cd}}^{+2}$ adsorption on cancrinite (CAN), a low-silica zeolite with a Si/Al ratio of 1.5.

### 3.4. Analysis of Structural Evolution in the Cd + ${\mathrm{CaCO}}_{3}$ → Ca + ${\mathrm{CdCO}}_{3}$ Reaction on the FAU Surface

**Figure 9:**
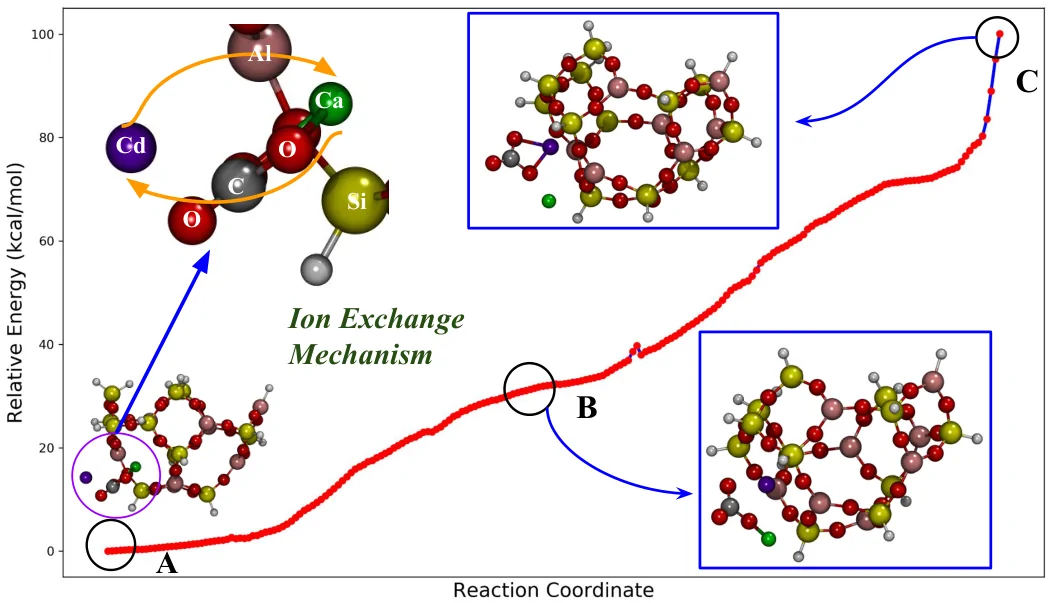
Reaction pathway for the Cd + ${\mathrm{CaCO}}_{3}$→ Ca + ${\mathrm{CdCO}}_{3}$ exchange on the FAU surface: (**A**) initial configuration with reactants (${\mathrm{CaCO}}_{3}$ and metallic Cd) interacting on the FAU surface; (**B**) intermediate state during the ion exchange process; and (**C**) final configuration following the formation of isolated Ca and a new ${\mathrm{CdCO}}_{3}$ unit.

As a concluding analysis, we performed analysis of the atomic coordinate changes for the Cd + ${\mathrm{CaCO}}_{3}$→ Ca + ${\mathrm{CdCO}}_{3}$ reaction where the results are presented in Figure 9. As illustrated, the process is predominantly endothermic with ΔE = 100.0 kcal/mol. The initial configuration where the reactants, metallic Cd and a ${\mathrm{CaCO}}_{3}$ unit, are in proximity on the FAU surface but have not yet undergone significant chemical change (see snapshot A). The Cd atom, located at a Cd–Ca distance of 4.197 Å, remains relatively isolated from the carbonate group at this stage. It is situated on the opposite side of the carbonate plane from the Ca atom, poised for interaction.

Snapshot B captures the system during the ion exchange process, where the Cd atom is beginning to integrate into the carbonate structure while the Ca atom is being displaced. The most significant observation is the simultaneous lengthening of the Ca-O interaction and shortening of the Cd-O interaction. The Ca-O distance has increased from 2.134 Å to 2.921 Å, an elongation of 0.79 Å that signifies the weakening and partial breaking of the calcium-carbonate bond. Concurrently, the Cd-O distance has decreased from 2.655 Å to 2.336 Å. While this change appears modest, the trajectory of the cadmium atom indicates it is moving into a bonding position relative to the carbonate oxygen atoms.

The carbonate geometry responds to the presence of the invading cadmium cation. The O-C-O angle has compressed from the ideal 120.2° to 115.8°, demonstrating that the carbonate electron density is polarizing toward the incoming Cd atom. The Ca-C distance has expanded from 2.559 Å to 2.863 Å, further evidence of calcium’s expulsion from the carbonate coordination sphere. The Cd-C distance has decreased from 3.589 Å to 2.788 Å, confirming that the cadmium is moving closer to the central carbon as it assumes its new position within the carbonate structure.

The third snapshot reveals the complete reorganization of the system into the products: a newly formed ${\mathrm{CdCO}}_{3}$ unit and an isolated calcium atom. The geometric parameters have undergone dramatic changes that reflect the different ionic radius and bonding preferences of cadmium compared to calcium. The carbonate group has substantially rearranged to accommodate the ${\mathrm{Cd}}^{2+}$ cation. The O-C-O angle has further decreased to 108.5°, a significant compression from both the initial 120.2° and the intermediate process 115.8°. The cadmium atom is now fully integrated into the carbonate structure. The calculated Cd-O distance to the nearest oxygen is 2.163 Å. At last, the calcium atom has been completely expelled from the carbonate. Its distance to the carbon atom is now 3.691 Å.

Since ${\mathrm{CdCO}}_{3}$ is less stable than ${\mathrm{CaCO}}_{3}$ under environmental conditions, the reverse reaction and subsequent release of ${\mathrm{Cd}}^{2+}$ may occur, especially when only traces of ${\mathrm{Cd}}^{2+}$ ions are present in solution. For relatively more concentrated solutions, there is a high probability that ${\mathrm{Ca}}^{2+}$ ions will be replaced by ${\mathrm{Cd}}^{2+}$ ions, an outcome observed experimentally, rather than the reverse, since the amount of released ${\mathrm{Ca}}^{2+}$ ions is insufficient to drive the reverse reaction. Similar observations were reported by Taylor [66], who compared the stability of ${\mathrm{CdCO}}_{3}$ with that of other substituents.

## 4. Conclusions

In this work, density functional theory calculations using the B3LYP-D3/def2-TZVPP level of theory were employed to investigate ${\mathrm{Cd}}^{2+}$ removal by ${\mathrm{CaCO}}_{3}$/FAU zeolite composites. Our analysis demonstrated that the most stable ${\mathrm{Na}}^{+}$ adsorption sites in the FAU framework are located at 12-membered rings containing two or three aluminum atoms, with binding energies of approximately −22 kcal/mol. This confirms that the local Al distribution and ring geometry govern cation stabilization via electrostatic interactions.

The ${\mathrm{Na}}^{+}$/${\mathrm{Cd}}^{2+}$ exchange reaction can proceed through a short-lived NaCd intermediate, which readily dissociates, enabling ${\mathrm{Cd}}^{2+}$ incorporation at anionic framework sites. The present findings also showed that dispersed ${\mathrm{CaCO}}_{3}$ units on the FAU surface yield ${\mathrm{Cd}}^{2+}$ binding energies ranging from −54 to −60 kcal/mol, which are significantly more favorable than those of aggregated configurations. This theoretical result corroborates previous experimental observations that dispersed carbonate particles enhance cadmium adsorption performance.

The calculated binding energies for ${\mathrm{Cd}}^{2+}$ on the ${\mathrm{CaCO}}_{3}$/FAU composite are substantially more negative than those reported for unmodified zeolites such as heulandite (−4.0 kcal/mol) and cancrinite (−6.81 kcal/mol), highlighting the superior performance of the modified adsorbent.

In summary, the current DFT modeling reliably identifies active sites, elucidates exchange pathways, and rationalizes performance enhancements in zeolite-based adsorbents. The results confirm that dispersed ${\mathrm{CaCO}}_{3}$ on FAU creates a more effective configuration for ${\mathrm{Cd}}^{2+}$ capture, providing a theoretical foundation for designing optimized heavy metal adsorbents. Although the present cluster model lacks explicit ${\mathrm{Na}}^{+}$ counter-cations, it captures the essential local interactions governing ion exchange. To improve quantitative predictions, future research will incorporate charge-compensating cations and different Si/Al ratios.

## Figures and Tables

**Table 1:** Average bond lengths and angles in the FAU Model.

Bond Type	Average Length (Å)
Si-O	1.684 ± 0.007
Al-O	1.712 ± 0.006
All T-O	1.689 ± 0.014
O-Si-O	109.4° ± 1.8°
O-Al-O	119.6° ± 2.3°

**Table 2:** Benchmark comparison of the computed HOMO–LUMO gap (ΔE) obtained in this work (B3LYP-D3/def2-TZVPP) with literature values for related zeolite clusters.

System	Methods	HOMO (eV)	LUMO (eV)	ΔE (eV)
This work	B3LYP-D3/def2-TZVPP	−7.99	−1.55	6.44
CuY Zeolite [46]	PBE/DZVP	-	-	2.26–2.68
Be-ERI Cluster [47]	B3LYP/6-31G(d,p)	−7.44	−0.49	6.95
Mg-ERI Cluster [47]	B3LYP/6-31G(d,p)	−7.60	−1.08	6.52
Ca-ERI Cluster [47]	B3LYP/6-31G(d,p)	−7.41	−1.17	6.24
Li-ABW [49]	PBEsol	-	-	5.08
$H_{12}$${\mathrm{Si}}_{12}$$O_{18}$($D_{2d}$) [45]	B3LYP/6-31G(d)	-	-	9.10
$H_{12}$${\mathrm{Si}}_{12}$$O_{18}$($D_{3d}$) [45]	B3LYP/6-31G(d)	-	-	9.14
${\mathrm{Ag}}_{3}$(${\mathrm{Si}}_{12}$$H_{12}$$O_{18}$) [50]	B3LYP/LANL2DZ	-	-	2.62
zeolite Y + ${\mathrm{Ni}}^{2+}$ [48]	B3LYP/Lanl2dz	-	-	2.45
zeolite Y + ${\mathrm{Ni}}^{2+}$ [48]	B3LYP/Lanl2dz	-	-	2.45
zeolite Y + ${\mathrm{Cu}}^{2+}$ [48]	B3LYP/Lanl2dz	-	-	1.36
zeolite Y + ${\mathrm{Cr}}^{2+}$ [48]	B3LYP/Lanl2dz	-	-	3.80
zeolite Y + ${\mathrm{Cd}}^{2+}$ [48]	B3LYP/Lanl2dz	-	-	5.44
zeolite Y + ${\mathrm{Pb}}^{2+}$ [48]	B3LYP/Lanl2dz	-	-	5.98
${\mathrm{Cs}}^{+}$/zeolite Y [51]	PW91/DNP	-	-	4.71

**Table 3:** Total energy differences (ΔE) and binding energies (BE) for ${\mathrm{Na}}^{+}$ adsorption at four sites in the FAU zeolite. ΔE values are given relative to the most stable configuration (**Site 2**).

Configuration	ΔE (kcal/mol)	BE (kcal/mol)
**Site 2**	0.00	−21.89
**Site 3**	0.57	−21.32
**Site 1**	13.50	−8.38
**Site 4**	18.58	−3.30

## Data Availability

The original contributions presented in this study are included in the article. Further inquiries can be directed to the corresponding author.

## Supplementary Materials

The following supporting information can be downloaded at https://www.mdpi.com/article/10.3390/jox16040133/s1, Figure S1: Optimized structures of a Na atom (blue) adsorbed on the faujasite surface at four distinct sites: (A) at an 8-membered ring containing two Al atoms, (B) at a 12-membered ring containing two Al atoms, (C) at a 12-membered ring containing three Al atoms, and (D) near an 8-membered ring containing a single Al atom; Figure S2: Optimized geometry of ${\mathrm{CaCO}}_{3}$ calculated at the B3LYP-D3BJ/def2-TZVPP level of theory; Figure S3: Adsorption configurations of a Cd atom coordinated with aggregated (A) one, (B) two, (C) three, and (D) four ${\mathrm{CaCO}}_{3}$ units within the FAU zeolite framework; Figure S4: Adsorption configurations of a Cd atom coordinated with dispersed (A) two, (B) three, and (C) four ${\mathrm{CaCO}}_{3}$ units within the FAU zeolite framework; Table S1: Vibrational frequencies, in ${\mathrm{cm}}^{-1}$, and energies for ${\mathrm{CaCO}}_{3}$.
