# Supplementary material for: A DFT Perspective on Cd2+ Removal by CaCO3/FAU Zeolite Frameworks: Bridging Theory and Experiment
Source: J Xenobiot. 2026 Jul 22;16(4):133. doi: 10.3390/jox16040133 (PMC13514745; doi:10.3390/jox16040133)
Supplement: Supplementary file 1 [file jox-16-00133-s001.zip › jox-4433589-supplementary-update.pdf]

Supplementary Materials: A DFT Perspective on Cd^{2+} Removal by $CaCO_3$/FAU Zeolite Frameworks : Bridging Theory and Experiment

Ramon S. da Silva, Mendelssolm K. Pietre, and Rodrigo G. Amorim

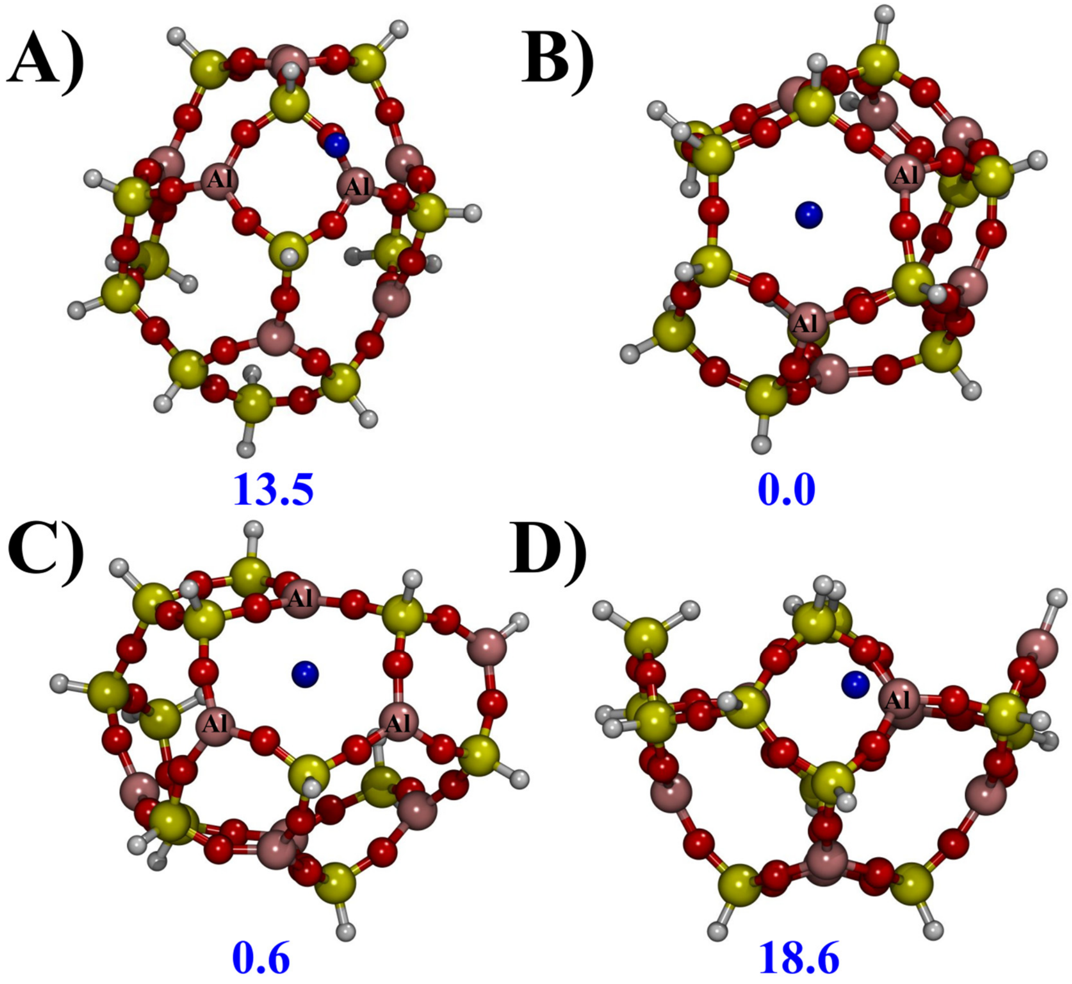

Figure S1. Optimized structures of a Na atom (blue) adsorbed on the faujasite surface at four distinct sites: A) at an 8-membered ring containing two Al atoms, B) at a 12-membered ring containing two Al atoms, C) at a 12-membered ring containing three Al atoms, and D) near an 8-membered ring containing a single Al atom.

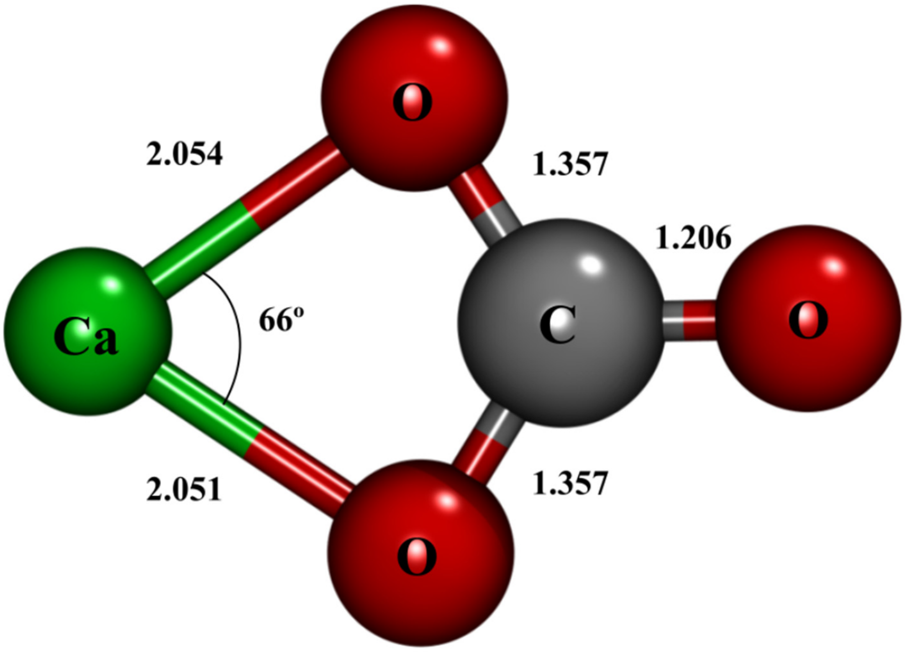

Figure S2. Optimized geometry of $CaCO_3$ calculated at the B3LYP-D3BJ/def2-TZVPP level of theory.

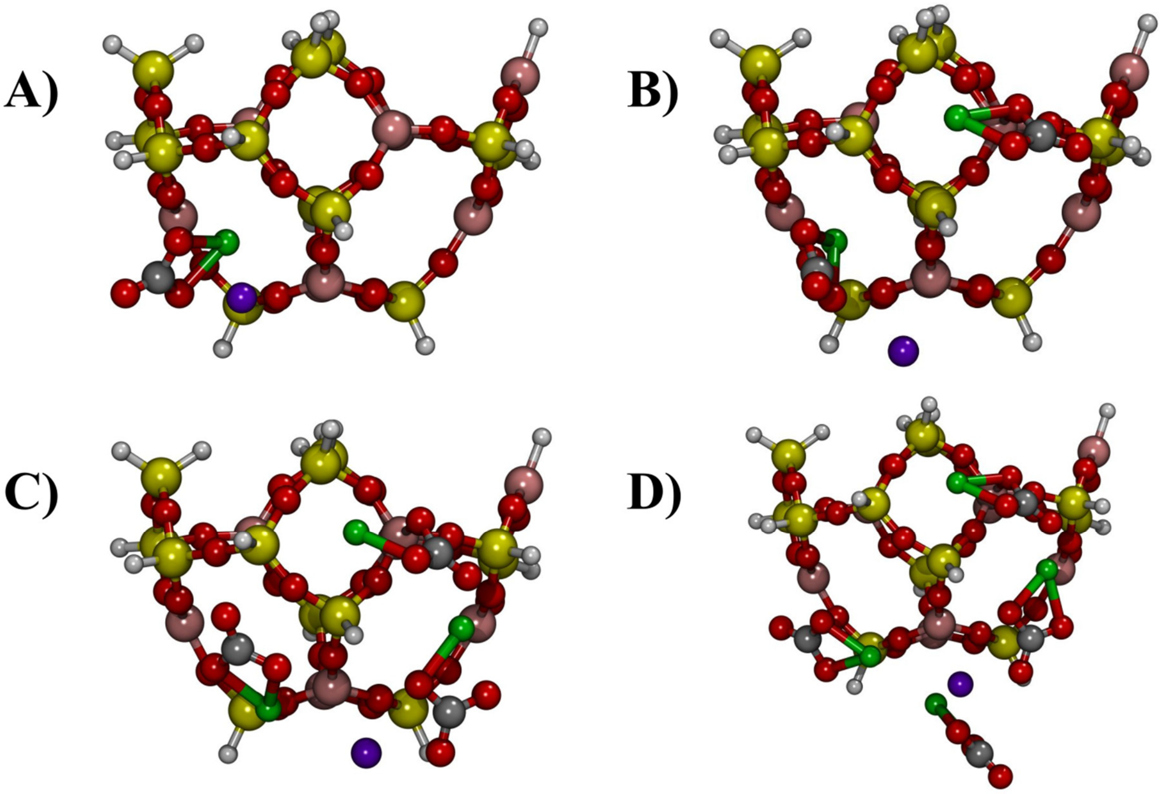

Figure S3. Adsorption configurations of a Cd atom coordinated with aggregated A) one, B) two, C) three, and D) four $CaCO_3$ units within the FAU zeolite framework.

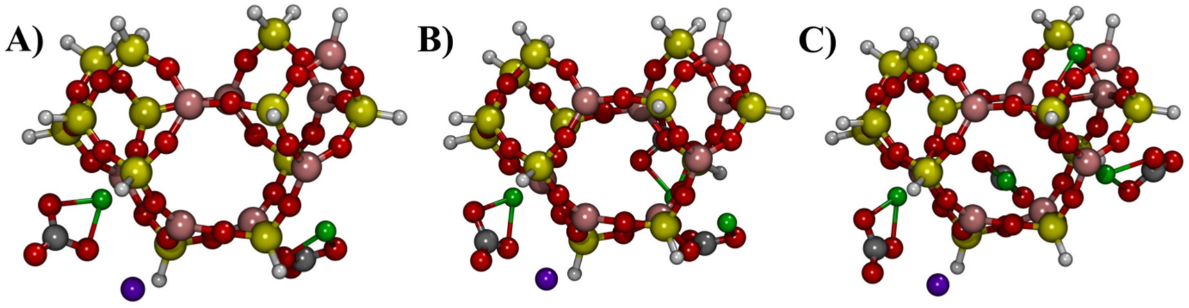

Figure S4. Adsorption configurations of a Cd atom coordinated with dispersed A) two, B) three, and C) four $CaCO_3$ units within the FAU zeolite framework.

Table S1.Vibrational frequencies, in cm^{-1}, and energies for $CaCO_3$.

This work	Exp. [1]	Exp. [2]
118	-	-
371	415	-
452	437	-
660	577	-
763	713	713
827	856	855
953	873	873
1080	1083	1082
1767	1794	1788

References

1. Gupta, U.; Singh, V.K.; Kumar, V.; Khajuria, Y. Experimental and theoretical spectroscopic studies of calcium carbonate (CaCO3).Mater. Focus 2015, 4, 164–169.
2. Kleiner, O.; Ramesh, J.; Huleihel, M.; Cohen, B.; Kantarovich, K.; Levi, C.; Polyak, B.; Marks, R.S.; Mordehai, J.; Cohen, Z.; et al. A comparative study of gallstones from children and adults using FTIR spectroscopy and fluorescence microscopy. BMC Gastroenterol. 2002, 2, 3.
